# Supplementary material for: Immune-Related Thyroiditis as a Predictor for Survival in Metastatic Renal Cell Carcinoma
Source: Cancers (Basel). 2022 Feb 10;14(4):875. doi: 10.3390/cancers14040875 (PMC8870210; doi:10.3390/cancers14040875)
Supplement: Supplementary file 1 [file cancers-14-00875-s001.zip › cancers-1550042-supplementary.pdf]

cancers

MDPI

Supplementary Material

Immune-Related Thyroiditis as a Predictor for Survival in Metastatic Renal Cell Carcinoma

Shira Sagie, Moran Gadot, Meital Levartovsky, Hadas Gantz Sorotsky, Raanan Berger, Michal Sarfaty and Ruth Percik

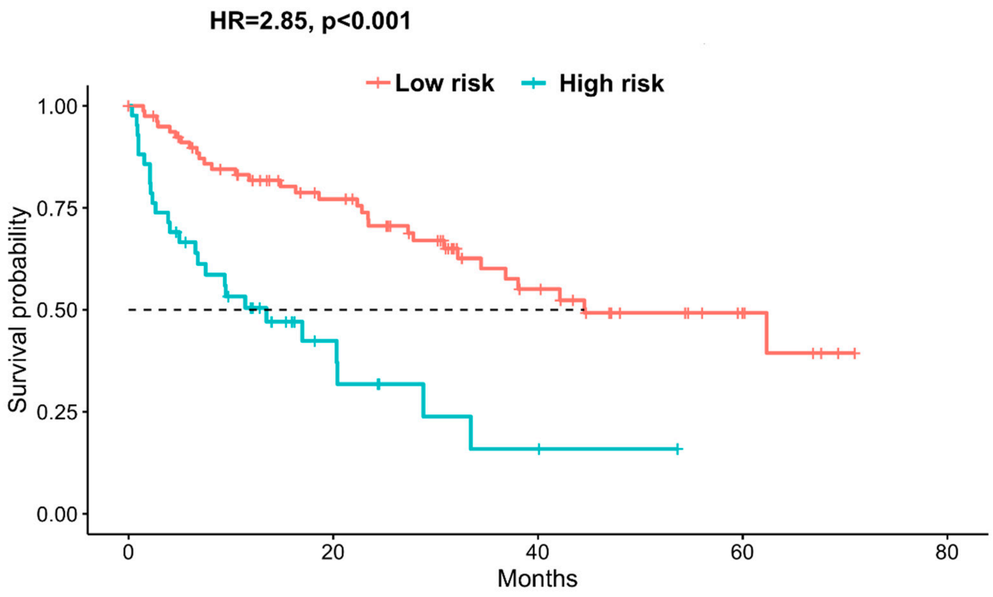

Figure S1. Survival probability from CPI treatment initiation by new risk groups. Kaplan-Meier plot of overall survival since CPI treatment intiation. HR and *p*-values calculated by Cox proportional hazard regression models.

Table S1. Immune related adverse events prevalence by treatment with a dual immunotherapy regimen (Ipi-Nivo) or not.

Variables	Overall (*n*, %)	Not treated by Ipi-Nivo	Treated by Ipi-Nivo	*p*
n	123	63	60	
Immune related thyroiditis	47 (38.2)	25 (39.7)	22 (36.7)	0.874
Immune related adverse events, any	57 (47.1)	24 (38.7)	33 (55.9)	0.086
Encephalitis	2 (1.6)	1 (1.6)	1 (1.7)	1.000
Pruritus	9 (7.3)	3 (4.8)	6 (10.0)	0.442
Rash	9 (7.3)	4 (6.3)	5 (8.3)	0.939
Neuropathy	3 (2.4)	1 (1.6)	2 (3.3)	0.966
Nephritis	6 (4.9)	5 (7.9)	1 (1.7)	0.232
Arthritis or myositis	4 (3.3)	1 (1.6)	3 (5.0)	0.577
Hepatitis	15 (12.2)	6 (9.5)	9 (15.0)	0.514
Pneumonitis	7 (5.7)	2 (3.2)	5 (8.3)	0.398
Diarrhea or colitis or gastritis	15 (12.2)	4 (6.3)	11 (18.3)	0.079
Hypoadrenalism	3 (2.4)	1 (1.6)	2 (3.3)	0.966
Treated by high dose steroids	37 (30.1)	17 (27.0)	20 (33.3)	0.568

Cancers **2022**, *14*, 875. https://doi.org/10.3390/cancers14040875 www.mdpi.com/journal/cancers
